# Well-Being Economies: A Harder but Still Important Health Advocacy Goal; A Response to Recent Commentaries

**DOI:** 10.34172/ijhpm.9311

**Published:** 2025-07-28

**Authors:** Ronald Labonté

**Affiliations:** School of Epidemiology and Public Health, University of Ottawa, Ottawa, ON, Canada.

## Background

 Since my article, and the four commentaries on it, were written, the election of Trump’s second presidency has thrown the neoliberal capitalist global order into chaos. No one knows quite yet what the illiberal regime the new Trump administration is dictatorially imposing will become, apart from the USA losing its status as a democracy and joining the global and growing ranks of autocratic rulers. This bodes ill for the goal of advancing well-being economies, a point on which I think all four commentators would likely agree. But this does not negate the importance of powerful counter-narratives to what two astute political writers, Naomi Klein and Astra Taylor, describe as the rise of “end times fascism” in the USA, a reference to the role played by religious extremists supporting the new regime and their belief that it is heralding the return of the Messiah.^1^ We need a compelling (and readily grasped) “better times well-being economy” along the lines of *buen vivir* and eco-socialist economics referenced in my article.^2^

 Brand-Correa begins her commentary with a dose of optimism about the prospect of doing so, arguing that the recent experience of the COVID-19 pandemic that I characterized as a global failure (in terms of equity) nonetheless offers “proof that rapid and dramatic change is possible” (in terms of rapid development of vaccines).^3^ The pandemic also “forced us to reflect on what we really need” as individuals and societies. It is hard to disagree with this sentiment. But will the necessity to transform capitalism into a different political economy engender the same sense of urgency as the pandemic? Especially since there is a new urgency created by Trump’s second presidency, in which the USA has become militarily belligerent and anti-global under a misogynist and racist narcissist?

 My rhetorical questions bear also on Brand-Correa’s enthusiasm for Universal Basic Services that are non-market based, which can decouple us somewhat from the planet destroying growth/consumption treadmill. Implementing or strengthening such services is challenged by its reliance on public taxation of private economic activities (profit, income, wealth) which, Brand-Correa notes, would be less than is often assumed since people will be healthier thanks to the services. Perhaps, but that assumes a direct relationship between health status and health care usage and cost. My preferred strategy would be to tax the hell out of the things we do not want and that are economically bad for well-being, such as excess wealth, income inequalities, extractive industries and unhealthy commodities. This would “lower the ceilings” of consumption which, consistent with degrowth economics, must decline if we are to survive as a species. As her commentary points out, most well-being economy policies described in my article focus more on “lifting floors” to reduce deprivations, important but insufficient from the vantage of planetary health.

 Hensher’s commentary cuts to the quick of this by identifying what distinguishes a radical well-being economy agenda from its window-dressing performativity: Is the economy seen as a necessary input to human and planetary well-being? Or is well-being argued important as a useful condition for continued economic growth?^4^ Both framings are likely to be found in any political statement about well-being economies, though the predominant tilt is likely easy to disinter. McLaren’s commentary does this with her reflection on Canadian public health policy discussions, in which promoting a “well-being economy” quickly truncates to promoting “well-being,” and any trenchant economic analyses (heterodox or otherwise) is rendered invisible.^5^ Hensher makes another important point when agreeing with my contention that the focus on measurement (the “beyond gross domestic product” career path for many indicator-driven researchers) is misplaced. Emphasis, instead, he argues, should go to transforming institutions to accept the transformational necessity of subordinating economic goals to people’s and planetary needs.

 He also adds a further opponent to such a transformation beyond only those with vested economic interests in retaining capitalism’s growth/consumption engine: the cult (or so I would describe it) of a “Nietzschean kick-back” which sees the equity and caring goals of well-being economics as “succouring the weak” and undermining the heroism and pursuit of self-excellence of the Übermenschen (supermen) as humanity’s primary goal. The current rise of autocratic rulers, for whom absolute power is excellence, and the technofeudalist oligarchs, for whom absolute wealth is excellence, aligns with this caution, although without the apparent willingness of such all-defining individuals to risk their own lives (their “heroism”) to achieve such goals. It also leaves the open question: Is it not capitalism that succours the Übermenschen?

 Legge joins the other commentators in adding something useful to my article’s analysis by identifying imperialism as a feature of capitalism with implications for how we might advance action on well-being economics.^6^ Imperialism entraps countries of the Global South (most of which are low- and middle-income by World Bank metrics) in an exploited dependency on the “imperium” of the Global North (today’s high-income and continuously colonizing nations, by one means or another). His argument is particularly apt given Trump’s presidential return. Some of the new US administration’s actions are forthrightly imperialist in the old expansionist sense, such as Trump’s repeated threats to take over Canada, Greenland, and Panama. Much of its imperialism, however, is simply using the economic weight of the USA, the role of its dollar as global reserve currency and the unilateral imposition of tariffs to bully countries into agreements that favour the interests of Trump and US-based transnational corporations. That Trump’s administration appears to ignore any rule of law with which it disagrees (domestic or international) reduces the emerging global order to one in which the only operating principle is a stripped-down old imperialism of “might is right.”

 Legge’s discussion of the 1974 New International Economic Order and its 2014 rebirth importantly reminds us that radical well-being economies must be global in reach to counter the historic and continuing exploitations of economic dependency. The rise of the BRICS+ (Brazil, Russia, India, China, South Africa, Egypt, Ethiopia, Indonesia, Iran, and the United Arab Emirates) group of countries with their aggregate economic power and some of their intended policy directions offers a potential non-compliant counterweight to the US hegemonic order. However, as Legge himself points out, this is weakened by the economic and political diversity of BRICS+ member states and by many of these countries’ leaders engaging in the same autocratic rule as that of the USA.

 McLaren, like other commentators, sees the appeal of a well-being economy and cites a number of real or hypothetical examples of such localized economics-in-action.^5^ Her primary and Canadian-focused argument is that promoting such an economy has so far been relatively absent from public health activism. Since she posted her commentary, Canada re-elected a centrist Liberal federal government on the basis of its promised “arms up” (a hockey term for defensive action) against the Trump administration’s repeated threats to Canadian sovereignty. But surprising to many who voted in the new government on this promise, it has since enacted this defensiveness by embracing fossil fuels, increasing military spending and rescinding its digital services tax opposed by Trump (to the benefit of the US tech oligarchs), all to negotiate a new trade deal to eliminate Trump’s punishing tariffs of questionable legality. This may spark more public health attention to the need for wholesale economic transformation.

 Reprising Brand-Correa’s opening optimism, McLaren concludes with the challenge on which all four commentators would likely agree:

 “We can passively await the impending political economy of authoritarian capitalism or worse, or we can try to meaningfully engage with a vision of an alternative—such as a well-being economy—that centres all people, all living things, and our planet through premises such as solidarity, cooperation, respect, and humility. Public health communities have an important decision to make.”

 Hensher, in his answer my article’s question, “can a well-being economy save us?” adds one important caveat:

 “Only if it can remain within the trajectory of declining material resource consumption and pollution that is actually required to return us to safety within planetary boundaries. We should surely aspire to the profoundly positive vision of conviviality and public abundance; but we ignore inconveniently hard ecological limits at our peril.”

## Ethical issues

 Not applicable.

## Conflicts of interest

 Author declares that he has no conflicts of interest.
